# Structural Design Calculation of Basalt Fiber Polymer-Modified RPC Beams Subjected to Four-Point Bending

**DOI:** 10.3390/polym13193261

**Published:** 2021-09-24

**Authors:** Yafeng Gong, Jianxing Yang, Xin He, Xiang Lyu, Hanbing Liu

**Affiliations:** 1 Department of Road and Bridge Engineering, Nanling Campus, College of Transportation, Jilin University, Changchun 130025, China; gongyf@jlu.edu.cn (Y.G.); jxyang20@mails.jlu.edu.cn (J.Y.); lvxiang18@mails.jlu.edu.cn (X.L.); lhb@jlu.edu.cn (H.L.); 2 China Construction Second Engineering Bureau Ltd., Beijing 100160, China

**Keywords:** construction materials, basalt fiber polymer-modified reactive powder concrete, cracking moment, normal section bending bearing capacity

## Abstract

In this paper, a basalt fiber surface was treated with coupling agent KH-550 and hydrochloric acid, and the basalt fiber polymer-modified active powder concrete (RPC) material was prepared. There are significant differences in material composition and properties between basalt fiber polymer-modified RPC and ordinary concrete, and the structural design calculation (cracking moment and normal section bending bearing capacity) of an ordinary reinforced concrete beam is no longer applicable. Thus, mechanical parameters such as displacement and strain of reinforcement basalt fiber polymer-modified RPC beams subjected to four-point bending were tested. The excellent compressive and tensile strengths of basalt fiber polymer-modified RPC were fully utilized. The tensile strength of basalt fiber polymer-modified RPC in the tensile zone of the beam was considered in the calculation of normal section bending bearing capacity of reinforcement basalt fiber polymer-modified RPC beams. The results showed that the measured values of the cracking moment and ultimate failure bending moment of reinforcement basalt fiber polymer-modified RPC beams were in good agreement with the calculated values. The established formulas for cracking moment and normal section bending bearing capacity can provide references for the design of reinforcement basalt fiber polymer-modified RPC simply supported beam and promote the wide application of basalt fiber polymer-modified RPC materials in practical engineering.

## 1. Introduction

Basalt fiber polymer-modified reactive powder concrete (RPC) is considered to be an innovative high property cement-based concrete material prepared by the theory of densified particle packing [1,2,3,4]. Basalt fiber polymer-modified RPC replaces the coarse aggregate in ordinary concrete with millimeter-level aggregate (quartz sand) and fills the gaps between quartz sand aggregates with micron-sized cementitious materials (cement) and submicron-sized cementitious materials (silica fume) [5,6]. In this way, the internal defect of basalt fiber polymer-modified RPC are controlled, and both the density and homogeneity are improved. Besides, basalt fiber polymer-modified RPC composites are cured by heat treatment and blended with high-performance, acid and alkali-resistant basalt fibers [7]. Owing to this, the microstructure of basalt fiber polymer-modified RPC materials is more compact and the mechanical properties, durability and toughness of basalt fiber polymer-modified RPC composites are improved. Therefore, basalt fiber polymer-modified RPC has the characteristics of ultra high strength, high durability and high temperature adaptability [8,9,10]. Basalt fiber polymer-modified RPC can effectively reduce the self-weight of structures and increase the spanning capacity, which has a broad application prospect in various infrastructure construction. At present, the engineering application of basalt fiber polymer-modified RPC materials is relatively few, mainly due to the lack of corresponding structural design calculation methods, while the calculation of cracking moment and normal section bending bearing capacity is an important part of structural design.

From the present research, it can be seen that researchers have proposed different methods for calculating cracking moments and bending bearing capacity for RPC structures and composite structures of RPC with other materials [11,12]. Zingaila et al. [13] experimentally investigated the mechanical properties of a combined beam made of ordinary concrete and ultra-high performance concrete and calculated the cracking moment of the combined beam using the layered method. Zhang et al. [14] conducted flexural tests on damaged bridge decks reinforced by ultra-high performance concrete and established analytical equations for the cracking and ultimate flexural bearing capacity of the composite structure. Yang et al. [15] proposed an analytical method for predicting the bending response of ultra-high-performance fiber-reinforced concrete structures, which can accurately predict the bending strength of ultra-high-performance fiber-reinforced concrete beams. Ujike et al. [16] used RPC to strengthen a part of the tensile zone of reinforced concrete beams and used elasticity theory to estimate the cracking moment of this composite material. Turker et al. [17] also researched the bending performance of ultra-high-performance fiber-reinforced concrete beams by four-point bending tests and proposed two numerical methods for predicting the nominal moment bearing capacity of these materials. Sim et al. [18] proposed flexural design guidelines for precast prestressed concrete members and found that the traditional equivalent rectangular stress block in compression can still be used to produce satisfactory results in prestressed concrete members. Prem et al. [19] developed an integrated nonlinear fracture mechanics model to predict the moment carrying capacity of ultra-high performance concrete reinforced damaged reinforcement concrete composite beams. Based on the planar section assumption and specific damage criterion, Guo et al. [20] used the numerical integration method to simulate the full process of flexural behavior of RPC and ordinary concrete composite beams and obtained the full section moment-curvature curves for different design scenarios. Chi et al. [21] investigated the effect of different water-cement ratios and different fiber compositions on the flexural performance of RPC beams using finite element analysis and deduced the formulae for calculating the flexural bearing capacity of RPC beams with different fiber compositions. Similarly, Chen et al. [22] derived an equation for calculating the ultimate flexural bearing capacity of ultra-high performance concrete beams utilizing ANSYS finite element simulation analysis. Qi et al. [23] carried out an experimental and analytical study of steel-ultra-high performance fiber concrete composite beams and calculated the flexural strength of the specimens using a simplified analytical method. Hasgul et al. [24] proved through an experimental study that the simplified numerical method for the flexural design of fiber-reinforced concrete was also applied to ultra-high-performance fiber-reinforced concrete beams. Cao et al. [25] investigated the flexural behavior of prestressed RPC in-situ panels suffering from four-point bending and established a modified formula for the flexural bearing capacity of prestressed RPC in-situ panels, considering the tensile strength of the RPC and prestressing stress. In addition, Cao et al. [26] also analyzed the effect of longitudinal reinforcement ratio and reinforcement diameter on the cracking moment of high-strength reinforcement RPC beams, and established formulas for calculating the resistance coefficient of plasticity in section and the cracking moment of high-strength reinforcement RPC beams. 

Existing research shows that there is a trend to develop concrete composite materials such as fiber-reinforced cementitious matrix and steel reinforced grout [27,28]. The basalt fiber polymer-modified RPC composites studied in this paper belong to the fiber-reinforced cement matrix. The basic mix proportion design, mechanical properties and durability of basalt fiber polymer-modified RPC composites have been discussed in previous studies of this research group. This paper focuses on the structural design calculation equations of basalt fiber polymer-modified RPC simply supported beams.

Basalt fiber polymer-modified RPC is a new material and differs significantly from other concrete materials in terms of material composition and properties. The formulae for calculating cracking moment and normal section bending bearing capacity of other concrete materials are not fully applicable to basalt fiber polymer-modified RPC. This paper thus investigated the mechanical properties of reinforcement basalt fiber polymer-modified RPC simply supported beams subjected to four-point bending. Based on the test data, the calculation methods of cracking moment and normal section bending bearing capacity were proposed for reinforcement basalt fiber polymer-modified RPC beam to provide a reference for the structural design and specification of reinforcement basalt fiber polymer-modified RPC beams.

## 2. Experimental Details

### 2.1. Materials and Mixture

**Figure 1:**
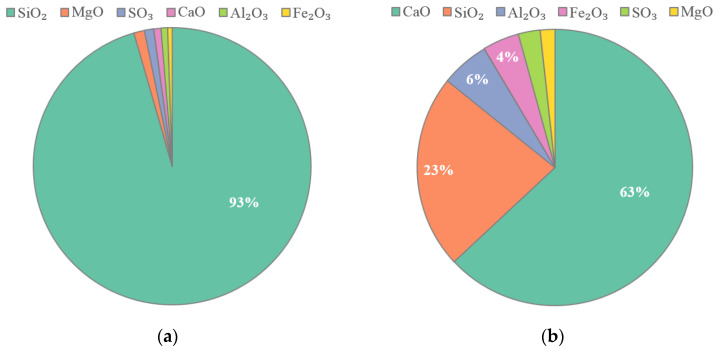
Chemical composition of cementing materials: (**a**) Silica fume; (**b**) Cement.

The material used to prepare Basalt fiber polymer-modified RPC beams consists of quartz sand, quartz powder, cement, silica fume, water reducer, basalt fiber, coupling agent, hydrochloric acid, water, and reinforcement. Three kinds of quartz sand with the sizes of 20~40 mesh, 40~80 mesh, and 80~120 mesh produced by the Zhenxing quartz sand factory (Luoyang, China) were used. These quartz sands were mixed in the ratio of 2:2:1 and filtered with square hole sieves of 0.6 mm, 0.3 mm, and 0.15 mm in order. The specification of quartz powder was 400 mesh. The silica fume SF92 produced by Jilin Changchun Si-Ao Technology Co., Ltd. (Jilin, China) and silicate cement P.II 52.5 produced by Jilin Yatai Cement Co., Ltd. (Jilin, China) were adopted. The chemical composition of silica fume and cement was determined by X-ray fluorescence spectrometer (manufactured by HORIBA Jobin Yvon, Paris, France) according to the “Method of Chemical Analysis of Silicate Rocks, Part 28: Determination of 16 Major and Minor Element Quantities” (GB/T 14506.28-2010) [29], and the results are shown in Figure 1. The coupling agent KH-550 is produced by Nanjing Chuangshi Chemical Additives Co., Ltd. (Jilin, China). The mix proportion of basalt fiber polymer-modified RPC is shown in Table 1. This mix proportion is the best mix proportion obtained from the previous research results of the research group [30]. According to the specification “Method of Testing Cement-Determination of Strength” (GB/T 17671-1999) [31], the compressive strength and flexural strength of basalt fiber polymer-modified RPC with this mix proportion were tested by computerized full-automatic compressive and flexural one-piece testing machine (manufactured by Meters Testing Machine Factory, Tianjin, China). The loading rates of compressive strength and flexural strength are 2.4 kN/s and 0.05 kN/s, respectively. The compressive strength and flexural strength of basalt fiber polymer-modified RPC prepared by this mix proportion were 115 MPa and 17 MPa, respectively. 

### 2.2. Specimen Preparation and Experimental Procedure

**Figure 2:**
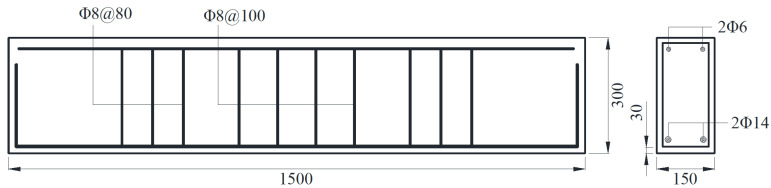
Dimension of the reinforcement basalt fiber polymer-modified RPC beam (mm).

In this paper, basalt fiber was first etched in hydrochloric acid, and then treated according to the ratio of KH-550 coupling agent to basalt fiber mass ratio of 3:100. Finally, three reinforcement basalt fiber polymer-modified RPC beams with different reinforcement ratios were prepared. The designed size of test beams was 1500 mm × 150 mm × 300 mm. Figure 2 shows the dimensions of three test beams and the relative positions of the rebar. 

The types and diameters of erective steel bars, stirrups, and main rebar of each test beam are shown in Table 2. In each test beam, two HPB300 steel bars with a diameter of 6 mm were used as erecting steel bars; ten HPB300 steel bars with a diameter of 8 mm were used as stirrups; two HRB335 rebar with a diameter of 12 mm, 14 mm, and 16 mm respectively were used as the main rebar. 

The prepared reinforcement basalt fiber polymer-modified RPC beams firstly were cured in 90 °C steam for 48 h. After curing, all test beams were performed the four-point bending test. The static response values (displacements, strains, etc.) of a reinforcement basalt fiber polymer-modified RPC beams subjected to four-point bending were recorded. Finally, considering the tensile strength of basalt fiber polymer-modified RPC in the tensile zone of the beam, the formulae for calculating the cracking moment and the normal section bending bearing capacity were established from the calculation principle applicable to the reinforcement basalt fiber polymer-modified RPC beam.

### 2.3. Experimental Setups

**Figure 3:**
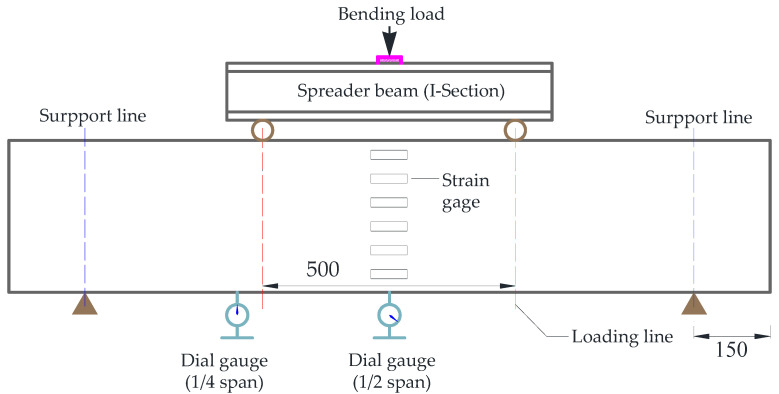
Sketch of the experimental setup for the four-point bending test (unit: mm).

The reinforcement basalt fiber polymer-modified RPC beam were tested with reference to the specification “Standard for Test Methods for Concrete Structures” (GBJ50152-2012) [32]. Loading method adopts four-point bending loading, loading device selection the WAW-1000 kN type electro-hydraulic servo universal testing machine produced by Jilin Jinli test technology Co., Ltd. (Jilin, China). The experimental setup is schematically shown in Figure 3. Displacement and strain were measured by dynamic and static strain testing machine DH3817 and static strain testing machine DH3818Y produced by Jiangsu donghua Test Technology Co. (Jiangsu, China). 

As shown in Figure 3, the dial gauges were arranged at the bottom of the 1/2 and 1/4 span of the beams for recording the deflection values during the loading process. Simultaneously, Six strain gauges were arranged along the beam height of 1/2 span to record the strain change during the test. Additionally, a multi-step loading process (20 kN for each step) was adopted, and the loading rate was controlled to be 0.037 kN/s.

## 3. Results

### 3.1. Test Results

Based on loads of the three reinforcement basalt fiber polymer-modified RPC beams at cracking and failure, the measured values of the cracking moment and ultimate failure bending moment of all test beams can be calculated as shown in Table 3.

### 3.2. Cracking Moment Calculation

#### 3.2.1. Calculation Principle of Cracking Moment

**Figure 4:**
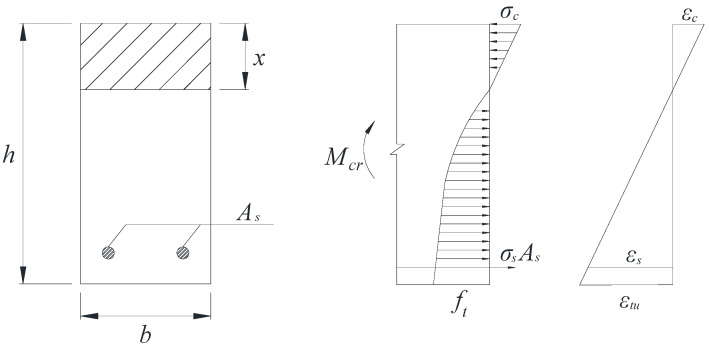
Calculation model of the cracking moment.

The cracking moment is the main index of cracking resistance and the foundation for the analysis of the mechanical properties of reinforcement basalt fiber polymer-modified RPC beams. The cracking moment is usually defined as the bending moment at the appearance of the first visible crack in the test beam. When the test beam is cracked, the tensile zone of the test beam shows a certain plastic deformation. The stress distribution graph is curved. The maximum stress in the tensile zone of the test beam reaches the ultimate tensile strength $f_{t}$, and the strain at the tensile edge reaches the ultimate tensile strain $\varepsilon_{tu}$. The basalt fiber polymer-modified RPC in the compression zone of the test beam is in the elastic phase, and the stress distribution graph is linear. The strain of basalt fiber polymer-modified RPC at the edge of the compressed zone is $\varepsilon_{c}$. In view of the above analysis, the calculation model of the cracking moment of the reinforcement basalt fiber polymer-modified RPC beam is shown in Figure 4.

According to the calculation model in Figure 4, Equations (1) and (2) can be derived. The cracking moment can be divided into two parts: the bending moment borne by the basalt fiber polymer-modified RPC matrix and the bending moment borne by the main rebars.

(1) $$M_{cr}=f_{t}\cdot W_{s},$$

(2) $$M_{cr}=M_{s}+M_{m},$$

where: $M_{cr}$ is the cracking moment of basalt fiber polymer-modified RPC; $f_{t}$ is the ultimate tensile strength of basalt fiber polymer-modified RPC; $W_{s}$ is the elastic–plastic resistance moment of the basalt fiber polymer-modified RPC beam section to the tensile edge; $M_{s}$ is the bending moment borne by the reinforcement; $M_{m}$ is the bending moment borne by the basalt fiber polymer-modified RPC matrix.

The section resistance moment plasticity influence coefficient γ was used to reflect the elasticplastic development degree in the tensile zone of the beam. The cracking moment formula is established by the principle of material mechanics. The formula for calculating the cracking moment is shown in Equation (3)

(3) $$M_{cr}=\gamma \cdot f_{t}\cdot W_{0},$$

where: γ is the section resistance moment plasticity influence coefficient; $W_{0}$ is the elastic-plastic resistance moment of the test beam’s converted section to the tensile edge.

Combining Equations (1) and (3), the formula of section resistance moment plasticity influence coefficient γ is shown in Equation (4).

(4) $$\gamma =\frac{W_{S}}{W_{0}},$$

#### 3.2.2. Calculation of $W_{s}$

**Figure 5:**
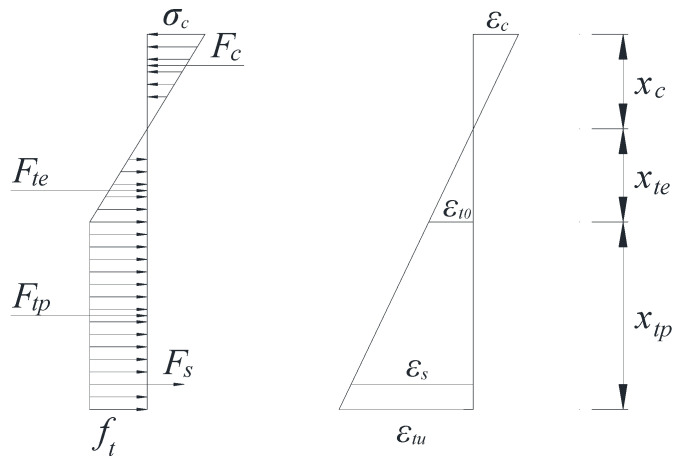
Simplified calculation model of normal section.

The stress distribution in the tensile zone was divided into the elastic and plastic tensile zones. The simplified calculation model of the normal section is shown in Figure 5. 

Based on test data, the flexural initial cracking tensile strains of Beam-1, Beam-2 and Beam-3 are 495.0 με, 491.8 με and 478.4 με, respectively. The ratios of the flexural initial cracking tensile strain to the peak tensile strain (213 με) of the basalt fiber polymer-modified RPC are 2.32, 2.31 and 2.25, respectively. The average value was taken as 2.29. Thus, $\varepsilon_{tu}=2.29\varepsilon_{t0}$ can be obtained. The combined force and bending moment of each part of the reinforcement basalt fiber polymer-modified RPC beam are shown in Equation (5) and Equation (6), respectively.

(5) $$\{\begin{array}{l} F_{c}=1.15\frac{x_{c}^{2}}{\left(h-x_{c}\right)}f_{t}b\\ F_{te}=0.22f_{t}b\left(h-x_{c}\right)\\ F_{tp}=0.56f_{t}b\left(h-x_{c}\right)\\ F_{s}=2.29\alpha_{E}f_{t}\rho bh_{0}\frac{h_{0}-x_{c}}{h-x_{c}} \end{array},$$

(6) $$\{\begin{array}{l} M_{c}=0.76f_{t}b\frac{x_{c}^{3}}{\left(h-x_{c}\right)}\\ M_{te}=0.07f_{t}b{\left(h-x_{c}\right)}^{2}\\ M_{tp}=0.4f_{t}b{\left(h-x_{c}\right)}^{2}\\ M_{s}=2.29\alpha_{E}f_{t}\rho bh_{0}\frac{{\left(h_{0}-x_{c}\right)}^{2}}{h-x_{c}} \end{array},$$

where: $F_{c}$ and $M_{c}$ are the combined force and bending moment of the beam in the compression zone; $F_{te}$ and $M_{te}$ are the combined force and bending moment of the beam in the elastic tension zone; $F_{tp}$ and $M_{tp}$ are the combined force and bending moment of the beam in the plastic tension zone; $F_{s}$ and $M_{s}$ are the combined force and bending moment of the main rebars; $x_{c}$ is the relative height of compression zone; b is the width of the beam section; h is the height of the beam section; $h_{0}$ is the effective height of the beam section; $\alpha_{E}$ is the section conversion factor; ρ is the reinforcement rate of the main rebars.

Equation (7) can be derived from the equilibrium condition of the force.

(7) $$F_{c}=F_{te}+F_{tp}+F_{s},$$

Bringing Equation (5) into Equation (7) to obtain Equation (8).

(8) $$x_{c}=\frac{-B+\sqrt{B^{2}-4AC}}{2A}$$

The parameters in Equation (8) are shown in Equation (9).

(9) $$\{\begin{array}{l} A={\left(a-1\right)}^{2}\\ B=(4ah-2h+2a^{2}\alpha_{E}\rho h_{0})\\ C=-\left(2ah^{2}-h^{2}+2a^{2}\alpha_{E}\rho h_{0}^{2}\right) \end{array}$$

Equation (10) can be obtained from the equilibrium conditions of moments.

(10) $$M_{cr}=bf_{t}\left[0.76\frac{x_{c}^{3}}{\left(h-x_{c}\right)}+0.47{\left(h-x_{c}\right)}^{2}+2.29\alpha_{E}\rho h_{0}\frac{{\left(h_{0}-x_{c}\right)}^{2}}{\left(h-x_{c}\right)}\right]$$

Combining Equations (1) and (10), the formula of $W_{s}$ is shown in Equation (11).

(11) $$W_{s}=b\left[0.76\frac{x_{c}^{3}}{\left(h-x_{c}\right)}+0.47{\left(h-x_{c}\right)}^{2}+2.29\alpha_{E}\rho h_{0}\frac{{\left(h_{0}-x_{c}\right)}^{2}}{\left(h-x_{c}\right)}\right]$$

#### 3.2.3. Calculation of $W_{0}$

**Figure 6:**
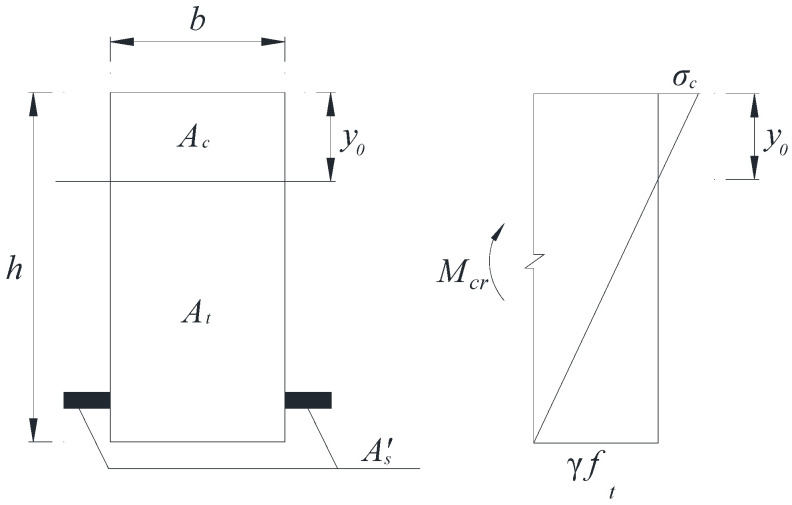
Conversion section and its stress distribution.

Using the equivalent conversion method, the main rebar area is equated to the basalt fiber polymer-modified RPC area with the same elasticity modulus. The converted section and its stress distribution are shown in Figure 6.

The conversion coefficient $\alpha_{E}$ for converting the main rebar area to the basalt fiber polymer-modified RPC area and the converted section area ${A^{\prime }}_{s}$ is shown in Equation (12) and Equation (13), respectively

(12) $$\alpha_{E}=\frac{E_{s}}{E_{c}}=\frac{1.95\times 10^{5}}{42.9\times 10^{3}}=4.55,$$

(13) $${A^{\prime }}_{s}=A_{s}\left(\alpha_{E}-1\right)=3.55A_{s},$$

According to the formula of material mechanics, the calculation of $W_{0}$ is shown in Equation (16).

(14) $$I_{0}=\frac{by_{0}^{3}}{3}+\frac{b{\left(h-y_{0}\right)}^{3}}{3}+3.55A_{s}{\left(h_{0}-y_{0}\right)}^{2},$$

(15) $$y_{0}=\frac{0.5bh^{2}+3.55A_{s}h_{0}}{bh+3.55A_{s}},$$

(16) $$W_{0}=\frac{I_{0}}{h-y_{0}}=\frac{by_{0}^{3}+b{\left(h-y_{0}\right)}^{3}+10.65A_{s}{\left(h_{0}-y_{0}\right)}^{2}}{3\left(h-y_{0}\right)},$$

#### 3.2.4. Calculations of γ and Cracking Moment

Taking Equations (11) and (16) into Equation (4), the section resistance moment plasticity influence coefficient γ of Beam-1, Beam-2 and Beam-3 can be calculated as 1.67, 1.71 and 1.75, respectively. It can be noticed that with the increase of reinforcement ratio, the section resistance moment plasticity influence coefficient γ increases gradually, which indicates that the increase of reinforcement ratio makes its plasticizing effect on the basalt fiber polymer-modified RPC around the reinforcement increase. 

The calculated value of cracking moment for all test beams can be obtained by bringing γ and Equation (16) into Equation (3) and comparing it with the test value of cracking moment, as shown in Table 4.

From Table 4, it can be observed that the ratio between the calculated and test values of the cracking moment has a small variation, and the mean value of this ratio is 1.01, the standard deviation is 0.05, and the variation coefficient is 0.05. The data error satisfies the requirements, which shows that the established formula for calculating the cracking moment of the reinforcement basalt fiber polymer-modified RPC beam is accurate.

### 3.3. Calculation of Normal Section Bending Bearing Capacity

#### 3.3.1. Basic Assumptions

Similar to the calculation of the normal section bending bearing capacity of the ordinary reinforced concrete beam, that of reinforcement basalt fiber polymer-modified RPC beam needs to adopt the plane section assumption in the process of reaching the limit state of bending bearing capacity. This can meet the error requirement of engineering calculation, and also can clarify the logic of the calculation process and the physical meaning of the calculation formula. For basalt fiber polymer-modified RPC materials, the standard value of tensile strength is significantly higher than that of ordinary concrete. Therefore, in this paper, the tensile strength of basalt fiber polymer-modified RPC in the tensile zone was considered in the calculation of the normal section bending bearing capacity of the reinforcement basalt fiber polymer-modified RPC beam.

#### 3.3.2. Equivalent Rectangular Stress Pattern

**Figure 7:**
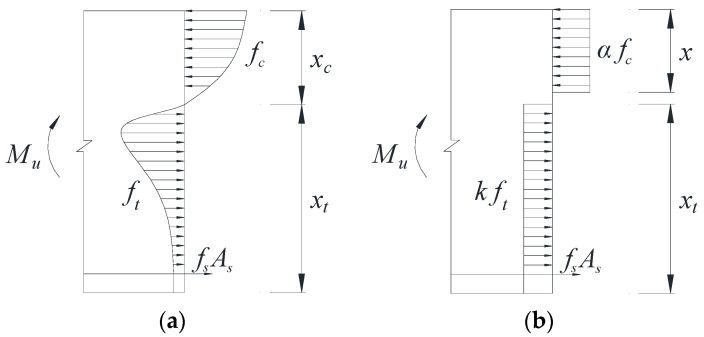
Stress distribution of normal section of properly reinforced beams under failure: (**a**) Actual stress distribution; (**b**) Equivalent stress distribution.

A rectangular stress diagram is used to equate the graphs of compressive and tensile stress curves in the cross-section of reinforcement basalt fiber polymer-modified RPC beam. The stress distribution at the time of damage of the reinforcement basalt fiber polymer-modified RPC beam normal section is shown in Figure 7.

According to the equivalent rectangular stress pattern of concrete in the compressed area, the combined compressive stress C and its distance $y_{c}$ from the neutral axis of concrete in the compressed area are shown in Equation (17) and Equation (18), respectively.

(17) $$C=\int_{0}^{x_{c}}\sigma_{c}\left(\varepsilon \right)\cdot b\cdot dy,$$

(18) $$y_{c}=\frac{\int_{0}^{x_{c}}\sigma_{c}\left(\varepsilon \right)\cdot b\cdot y\cdot dy}{\int_{0}^{x_{c}}\sigma_{c}\left(\varepsilon \right)\cdot b\cdot dy},$$

In accordance with the plane section assumption, the distance y from the neutral axis is related to the compressive strain of concrete at this location as in Equation (19).

(19) $$\frac{\varepsilon }{\varepsilon_{cu}}=\frac{y}{x_{c}},$$

Substitute Equation (19) into Equations (17) and (18) to obtain the resultant force *C* and its distance $y_{c}$ from the neutral axis is shown in Equation (20) and Equation (21) respectively.

(20) $$C=\frac{x_{c}\cdot b}{\varepsilon_{cu}}\cdot \int_{0}^{\varepsilon_{cu}}\sigma_{c}\left(\varepsilon_{c}\right)\cdot d\varepsilon ,$$

(21) $$y_{c}=\frac{x_{c}}{\varepsilon_{cu}}\cdot \frac{\int_{0}^{\varepsilon_{cu}}\sigma_{c}\left(\varepsilon \right)\cdot b\cdot \varepsilon \cdot d\varepsilon }{\int_{0}^{\varepsilon_{cu}}\sigma_{c}\left(\varepsilon \right)\cdot b\cdot d\varepsilon },$$

Let the maximum value of the combined compressive stress and its distance from the neutral axis be Equation (22) and Equation (23), respectively.

(22) $$\sigma_{cu}=\int_{0}^{\varepsilon_{cu}}\sigma_{c}\left(\varepsilon \right)d\varepsilon ,$$

(23) $$y_{cu}=\frac{\int_{0}^{\varepsilon_{cu}}\sigma_{c}\left(\varepsilon \right)\cdot \varepsilon \cdot d\varepsilon }{\int_{0}^{\varepsilon_{cu}}\sigma_{c}\left(\varepsilon \right)d\varepsilon },$$

Then the combined force *C* and its distance $y_{c}$ from the neutral axis are Equation (24) and Equation (25), respectively.

(24) $$C=x_{c}\cdot b\cdot \frac{\sigma_{cu}}{\varepsilon_{cu}},$$

(25) $$y_{c}=\frac{x_{c}}{\varepsilon_{cu}}\cdot y_{cu},$$

Let $k_{1}=\frac{\sigma_{cu}}{f_{c}\varepsilon_{cu}}$,$k_{2}=\frac{y_{cu}}{\varepsilon_{cu}}$, then the bending moment assumed by the basalt fiber polymer-modified RPC in the pressure zone is shown in Equation (26).

(26) $$M_{c}=C\cdot \left(h_{0}-x_{c}+y_{c}\right)=k_{1}f_{c}x_{c}b\left[h_{0}-\left(1-k_{2}\right)x_{c}\right],$$

Given the parameter α, the bending moment assumed by the basalt fiber polymer-modified RPC in the compression zone is given in Equation (27).

(27) $$M_{c}=\alpha f_{c}bx\cdot \left(h_{0}-\frac{\beta }{2}x\right),$$

Combining Equations (26) and (27) to obtain Equation (28).

(28) $$\{\begin{array}{l} \alpha =\frac{k_{1}}{2(1-k_{2})}\\ \beta =2(1-k_{2}) \end{array},$$

According to the basalt fiber polymer-modified RPC compressive stress–strain relationship showed in Equation (29), $k_{1}$ and $k_{2}$ were calculated to be 0.693 and 0.615, respectively. Then bring the $k_{1}$ and $k_{2}$ into Equation (28), α and β were calculated to be 0.92 and 0.76, respectively.

(29) $$\{\begin{array}{cc} y=1.12x+0.11x^{2}-0.65x^{6} & 0\leq x<1\\ y=1 & x\geq 1 \end{array},$$

Similarly, the curved tensile stress diagram of the tensile zone is equated to a rectangular tensile stress diagram, and the stress height $kf_{t}$ of the equivalent graph can be deduced backward from the equilibrium conditions and the test data. Equations (30) and (31) can be derived from the equilibrium conditions of forces and moments in Figure 7

(30) $$0.92f_{c}bx=f_{y}A_{s}+kf_{t}b\left(h-\frac{x}{0.76}\right),$$

(31) $$M_{u}=0.92f_{c}bx\left(h_{0}-\frac{x}{2}\right)-kf_{t}b\left(h-\frac{x}{0.76}\right)\left[0.5\left(h-\frac{x}{0.76}\right)-a_{s}\right],$$

The parameters b, h, $h_{0}$, α, β, $f_{c}$, $f_{y}$, $A_{s}$, $a_{s}$, and $f_{t}$ of Equations (30) and (31) are known quantities, and the relative compressive zone height x can be obtained by bringing the test data into Equations (30) and (31). The equivalent coefficient k can be calculated by bringing the obtained relative compressive zone height x into Equation (30). The calculated values of k for Beam-1, Beam-2, and Beam-3 are 0.32, 0.33, and 0.29, respectively. In order to simplify the calculation, the value of k for the reinforcement basalt fiber polymer-modified RPC beam is safely taken as 0.2.

#### 3.3.3. Calculation of Normal Section Bending Bearing Capacity

Combining Equations (30) and (31), and bringing in the value of k, the ultimate failure bending moment of the reinforcement basalt fiber polymer-modified RPC beam is obtained from the equilibrium condition in Equation (32).

(32) $$\{\begin{array}{l} 0.92f_{c}bx=f_{y}A_{s}+0.2f_{t}b\left(h-\frac{x}{0.76}\right)\\ M_{u}=0.92f_{c}bx\left(h_{0}-\frac{x}{2}\right)-0.2f_{t}b\left(h-\frac{x}{0.76}\right)\left[0.5\left(h-\frac{x}{0.76}\right)-a_{s}\right] \end{array},$$

The calculated values of the ultimate failure bending moment of each test beam can be calculated by Equation (32) and compared it with the test value of the ultimate failure bending moment, as shown in Table 5

From Table 5, it can be seen that the ratio between the calculated and tested values of the ultimate failure bending moment of the reinforcement basalt fiber polymer-modified RPC beams have a small variation, and the mean value of this ratio is 1.07, the standard deviation is 0.02, and the coefficient of variation is 0.02. The data error meets the requirements, which shows that the established formula for calculating the normal section bending bearing capacity of the reinforcement basalt fiber polymer-modified RPC beam is accurate.

#### 3.3.4. Relative Pressure Zone Height and Reinforcement Ratio Range

Using the data of the test beams in this paper and the parameters of the main rebar used into Equation (33), the relative height of compressive area $\xi_{b}$ is 0.48.

(33) $$\xi_{b}=\frac{\beta }{1+\frac{f_{y}}{\varepsilon_{cu}E_{s}}},$$

In order to ensure that the beam structure meets the moderate reinforcement ratio damage, it is also necessary to calculate the maximum and minimum reinforcement ratio of basalt fiber polymer-modified RPC to HRB335 main rebar.

The reinforcement rate of basalt fiber polymer-modified RPC simply supported beam is the maximum reinforcement rate when meets $\xi =\xi_{b}$. Bringing the test beam parameters and results into Equation (34) leads to a maximum reinforcement ratio of about 18% for HRB335 main rebar for basalt fiber polymer-modified RPC.

(34) $$\rho_{\max}=\frac{A_{s}}{bh_{0}}=\frac{\xi_{b}bh_{0}\left(0.92f_{c}+0.26f_{t}\right)-0.2f_{t}bh}{f_{y}bh_{0}},$$

The HRB335 reinforcement rate of the basalt fiber polymer-modified RPC beam is the minimum reinforcement rate when the bending moment limit value of the test beam is equal to the bending moment of the plain basalt fiber polymer-modified RPC beam with the same section without reinforcement when it is about to crack. Bringing the parameters and results of the test beam into Equation (35), the minimum reinforcement rate of HRB335 reinforced basalt fiber polymer-modified RPC is about 0.71%.

(35) $$\rho_{\min}=\frac{A_{s}}{bh_{0}}=\frac{\gamma f_{t}W_{0}-0.2f_{t}b\left(h-\frac{x}{0.76}\right)\left[0.5\left(h-\frac{x}{0.76}\right)+\frac{x}{2}\right]}{f_{y}\left(h_{0}-\frac{x}{2}\right)bh_{0}},$$

## 4. Discussion

**Figure 8:**
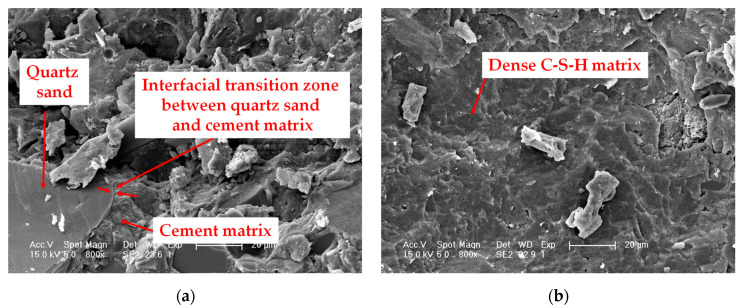

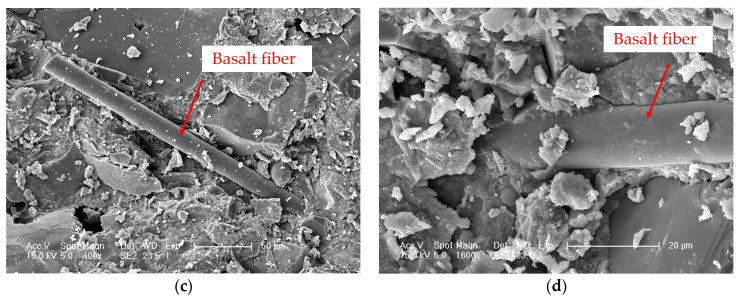
Microstructure of basalt fiber polymer-modified RPC: (**a**) Interfacial transition zone between quartz sand and cement matrix; (**b**) Dense C–S–H matrix; (**c**) Basalt fiber 400×; (**d**) Basalt fiber 1600×.

In this paper, basalt fiber treated with a coupling agent was used to modify the RPC material. Visualized scanning electron microscopy (SEM) was adopted to analyze the microstructure of basalt fiber modified polymer RPC, and the results are shown in Figure 8. Figure 8a shows that the thickness of the interfacial transition zone between the aggregate quartz sand and the cement matrix is negligible. Basalt fiber polymer-modified RPC replaces coarse aggregates in ordinary concrete with fine quartz sand, which eliminates mechanical, physical and chemical differences between normal concrete aggregates and mortar. As a result, the internal defects of basalt fiber polymer-modified RPC were reduced and the compactness was improved. From Figure 8b, it can be seen that the microstructure of basalt fiber polymer-modified RPC is denser and the hydration products are mainly dense C–S–H matrix. This is due to the fact that the basalt fiber polymer-modified RPC is cared for by heat treatment, which makes the hydration process more complete, changing the properties of the hydration products and reducing the porosity. It can be observed from Figure 8c that the basalt fibers are distributed in a random direction in the RPC material, and there is no agglomeration. Figure 8d reveals that the basalt fibers are tightly bonded to the cement matrix. These characteristics illustrate that basalt fibers form a tight monolith with the cement matrix, which can jointly withstand stress changes caused by external loads and temperature. The above-mentioned microstructural characteristics of basalt fiber polymer-modified RPC are important reasons for its excellent mechanical and durability properties.

It is owing to the excellent mechanical properties and durability of basalt fiber polymer-modified RPC composites that this study applies basalt fiber polymer-modified RPC to simply supported beams and investigates its structural design calculation equations. In contrast to ordinary concrete, the compressive and flexural strengths of basalt fiber polymer-modified RPC are substantially increased. Therefore, the flexural strength of concrete in the tensile zone needs to be considered in the design calculation of basalt fiber polymer-modified RPC simply supported beams. In this paper, from the calculation principle, the contribution coefficient of concrete in the tensile zone of basalt fiber polymer-modified RPC to the bending bearing capacity is 0.2 from the experimental data.

## 5. Conclusions

In this study, design calculations of basalt fiber polymer-modified RPC simply supported beams subjected to four-point bending were studied. Several conclusions derived based on the experimental results may be summarized as follows:

(1) The hydration products of basalt fiber polymer-modified RPC are mainly dense C–S–H matrix and the thickness of the interfacial transition zone between the aggregate quartz sand and the cement matrix is negligible.
(2) Basalt fibers treated with coupling agents in RPC materials are distributed in a random direction, and the basalt fibers are tightly bonded to the RPC matrix.
(3) The section resistance moment plasticity influence coefficient of the reinforcement basalt fiber polymer-modified RPC simply supported beam is 1.7; the relative height of the compressive area is 0.48; the minimum and maximum reinforcement ratios of HRB335 rebar are 0.71% and 18%, respectively.
(4) The established formulas for cracking moment and normal section bending bearing capacity is reasonably accurate. The research results of this paper can provide references for the design of reinforcement basalt fiber polymer-modified RPC simply supported beam and promote the wide application of basalt fiber polymer-modified RPC materials in practical engineering.

## Figures and Tables

**Table 1:** Mix proportion of basalt fiber polymer-modified RPC (kg/m^3^).

Water	Cement	Silica Fume	Quartz Sand		Quartz Powder	Basalt Fiber	Water Reducer
			0.15 mm~0.3 mm	0.3 mm~0.6 mm			
151.5	841.8	210.4	364.2	582.8	311.4	12	52.6

**Table 2:** Types and diameters of steel bars in test beams.

Teat Beam Number	Erecting Steel Bar		Stirrup		Main Rebar	
	Type	Diameter (mm)	Type	Diameter (mm)	Type	Diameter (mm)
Beam-1	HPB 300	6	HPB 300	8	HRB 335	12
Beam-2	HPB 300	6	HPB 300	8	HRB 335	14
Beam-3	HPB 300	6	HPB 300	8	HRB 335	16

**Table 3:** Measured values of the cracking moment and ultimate failure bending moment.

Teat Beam	Cracking Moment (kN·m)	Ultimate Failure Bending Moment (kN·m)
Beam-1	15.05	39.40
Beam-2	15.75	47.00
Beam-3	17.50	52.20

**Table 4:** Calculated and test values of the cracking moment.

Teat Beam	$\mathrm{Calculated}\;\mathrm{Value}\;M_{cr}^{c}$(kN·m)	$\mathrm{Test}\;\mathrm{Value}\;M_{cr}^{t}$(kN·m)	$M_{cr}^{c}$ $/M_{cr}^{t}$
Beam-1	15.78	15.05	1.05
Beam-2	16.23	15.75	1.03
Beam-3	16.73	17.50	0.96

**Table 5:** Calculated and test values of ultimate failure bending moment.

Teat Beam	$\mathrm{Calculated}\;\mathrm{Value}\;M_{u}^{c}$(kN·m)	$\mathrm{Test}\;\mathrm{Value}\;M_{u}^{t}$(kN·m)	$M_{u}^{c}$ $/M_{u}^{t}$
Beam-1	43.00	39.40	1.09
Beam-2	49.02	47.00	1.04
Beam-3	55.88	52.20	1.07

## Data Availability

Not applicable.
